# Parametric identification of the mathematical model of the micro-arc oxidation process

**DOI:** 10.1016/j.heliyon.2023.e19995

**Published:** 2023-09-10

**Authors:** Anatoliy Semenov, Ekaterina Pecherskaya, Pavel Golubkov, Sergey Gurin, Dmitriy Artamonov, Yuliya Shepeleva

**Affiliations:** a Department of Information and Measurement Equipment and Metrology, Penza State University, Krasnaya Street 40, Penza 440026, Russia; b Polytechnic Institute, Penza State University, Krasnaya Street 40, Penza 440026, Russia

**Keywords:** Micro-arc oxidation, Non-linear models, Parametric identification, Neural networks, Reservoir computing

## Abstract

The article is aimed at solving the problem of parametric identification of non-linear object models using the example of a mathematical model of the micro-arc oxidation process. An algorithm for parametric identification, based on an experiment in the micro-arc oxidation process, the results of which form a training and control sample is proposed; sequential training of neural networks and calculation of the parameters estimates of the nonlinear model according to experimental data are performed. Experimental testing of the proposed method of neural network parametric identification on the example of the micro-arc oxidation process confirmed that the standard deviation of current and voltage from the nominal values does not exceed ±4%. The obtained results were used in the development of an intelligent hardware-software complex for the production of protective coatings by the micro-arc oxidation method.

## 1 Introduction

The high physical and chemical properties of the coatings obtained in the micro-arc oxidation (MAO) process determine their wide application for protecting products from the adverse effects of the environment. The combination of high microhardness and wear resistance with corrosion resistance ensures the wide application of products with such coatings in many industries [[1], [2], [3]].

A large number of studies have been devoted to the improvement of the considered MAO process, among which one can note the possibility of imparting new properties to coatings by adding nanoparticles to the electrolyte [[4], [5], [6]], the search for new applications of MAO coatings [7,8], the improvement of their characteristics [[9], [10], [11], [12]], optimization [[13], [14], [15], [16], [17], [18]] and control [19,20] of this technological process.

A number of scientific studies are devoted to the modeling of technological processes using artificial intelligence [[21], [22], [23]], for example, the article [24] presents the results of using neural networks to simulate the erosion wear of WC-10Co4Cr coatings with the addition of yttrium oxide. Effective control of such a multidimensional and interrelated process combining chemical, electrochemical and plasma-chemical reactions is impossible without its deep study and the construction of a reduced mathematical model of the process that satisfies Akaike's criteria [25].

The analysis of various methods of parametric identification made it possible to dwell on recurrent methods that provide identification in real time with relatively simple computational algorithms and acceptable convergence of estimates. Carrying out identification allows to carry out current control, management and automation of the process, thereby increasing its technical and economic indicators.

## 2 Mathematical model of the micro-arc oxidation process

**Fig. 1:**
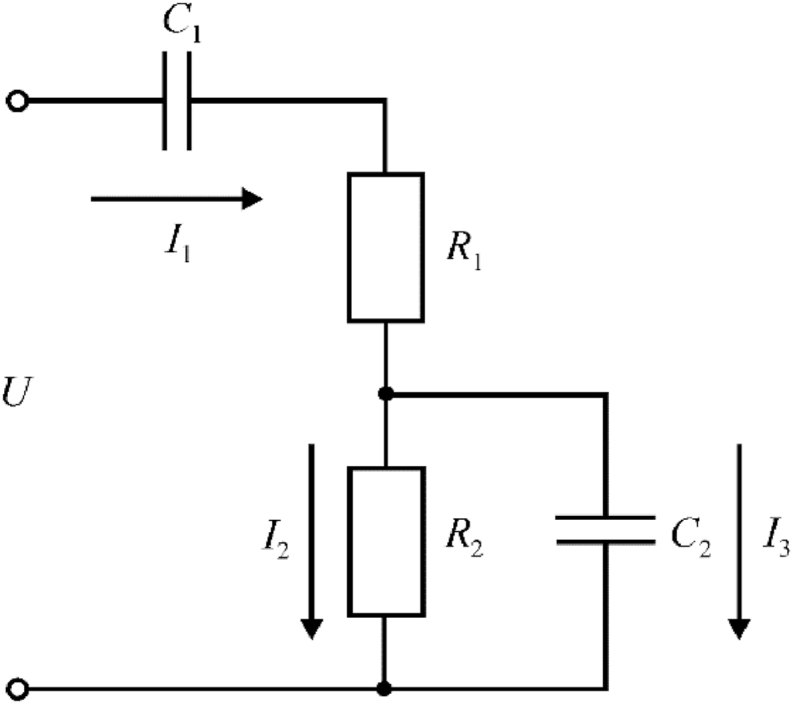
Equivalent electric circuit of micro-arc oxidation process: *U* – voltage; *I*_1_, *I*_2_, *I*_3_ – currents of the galvanic cell; *R*_1_ – electrolyte resistance; *C*_1_ – electrolyte capacitance; *R*_2_, *C*_2_ – non-linear active resistance and capacitance reactance of coating.

The studies carried out in Ref. [26] made it possible to develop a nonlinear electrophysical model of the oxidation process, which is shown in Fig. 1.

The electrolyte resistance is modeled by the active resistance *R*_1_, the cell coating resistance is modeled by a parallel connection of the non-linear active resistance *R*_2_ and the capacitance reactance *C*_2_. Capacitance *C*_1_ is connected in series with a galvanic cell for current limitation.

To test the adequacy of this model, experimental studies were carried out, during which oxide coatings were applied to 10 rectangular samples 23 × 15 × 1.5 mm in size from commercial aluminum grade AD31T1 using an automated capacitor-thyristor installation for micro-arc oxidation of our own design. The coatings were formed on a sinusoidal current with a frequency of 50 Hz in the anode-cathode mode with a current density of 10 A/dm g/l. An aqueous solution NaOH (0.5 g/l) with the addition of Na2SiO3 (80 g/l) was used as an electrolyte. During the MAO process, the oscillograms of the current and voltage drop in the galvanic cell were measured (two periods with a total duration of 40 ms, which corresponds to 256 points) every 60 s for 30 min. The measured values of current and voltage were saved to a text file. The received information was processed using the MATLAB mathematical calculation system.

**Fig. 2:**
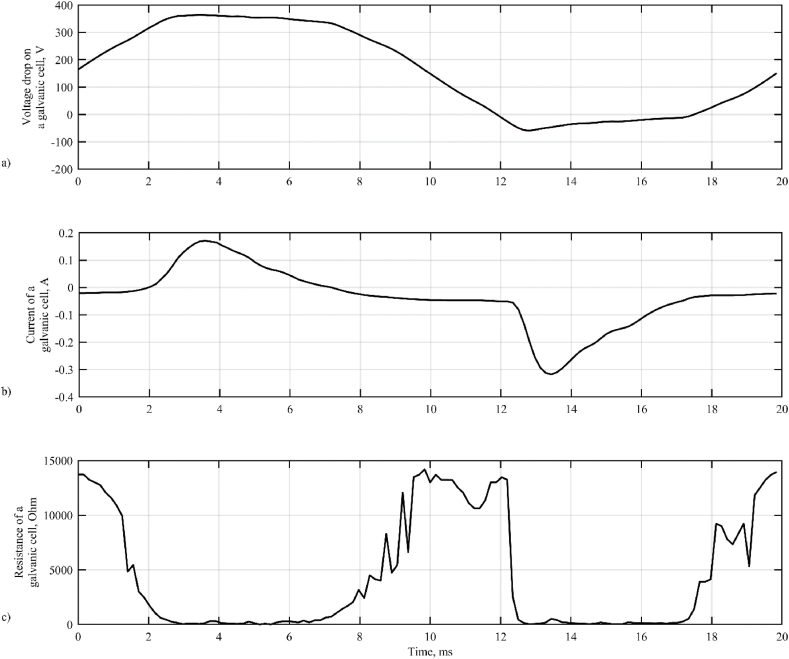
Oscillograms of voltage (a), current (b) and resistance (c) of a galvanic cell.

Fig. 2 shows the experimental oscillograms of the voltage drop *U*_1_ and current *I*_1_ on a galvanic cell recorded during one period of the supply voltage and the calculated oscillogram of the cell impedance, calculated as the ratio of the voltage drop increment to the current increment (Δ*U*_1_)/(Δ*I*_1_).

An analysis of the oscillograms allows to conclude that they contain four characteristic sections. In the first section (≈2–7 ms), the coating breaks down with a positive half-wave, the voltage applied to it, and the coating resistance drops sharply. In the second section (≈7–11 ms), the current in the cell changes polarity and the coating resistance is restored to its original value of approximately 12 kOhm. In the third section (≈11–17 ms), the coating is broken down by a negative voltage drop. In the fourth section (≈17–22 ms), the voltage drop across the cell changes sign and the coating resistance is restored.

Assuming that the electrolyte resistance *R*_1_ remains constant, on the order of several tens of ohms, we can assume that the coating resistance depends nonlinearly on the voltage applied to it and the current flowing through it.

**Fig. 3:**
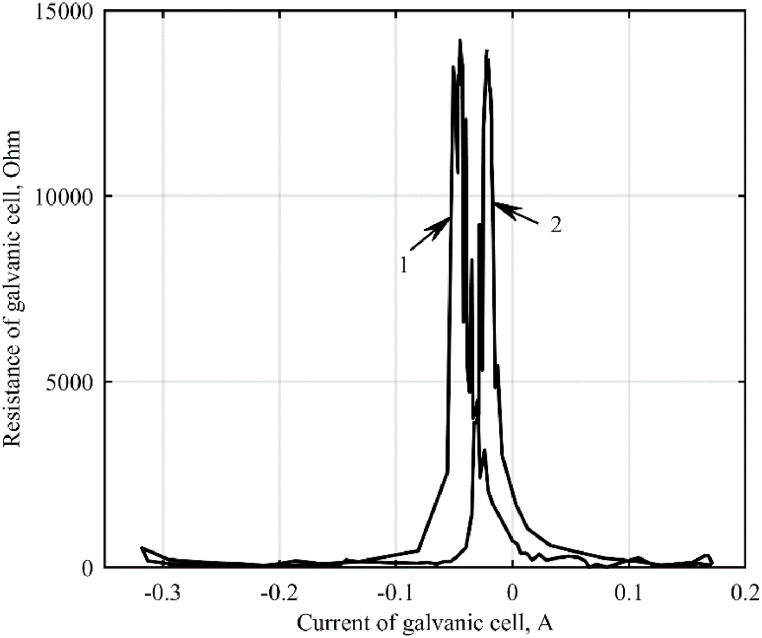
Dependence of the resistance of a galvanic cell on the current flowing through it: 1 – negative half-wave; 2 – positive half-wave.

**Fig. 4:**
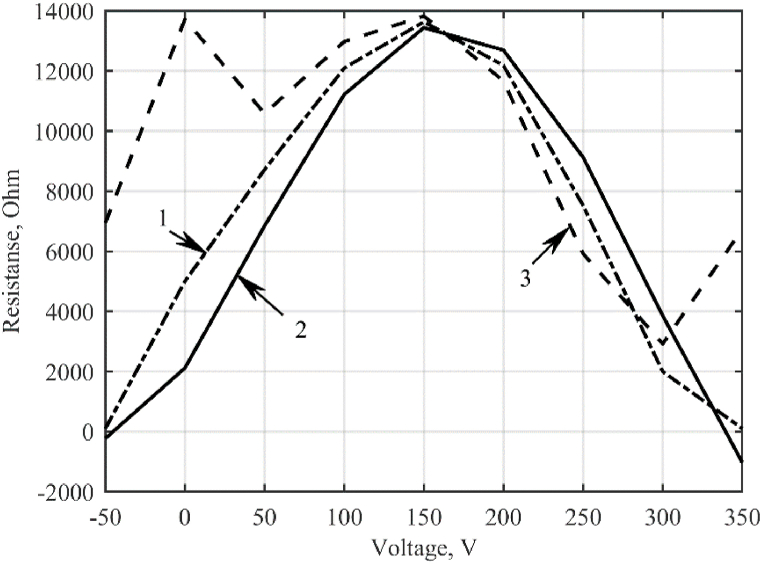
Dependences of the cell resistance on the voltage applied to it: 1 – fitting curve; 2 – positive half-wave; 3 – negative half-wave.

These dependences calculated from experimental oscillograms for positive and negative cell voltage drops are shown in Fig. 3, Fig. 4.

Due to the ambiguity of the resistance dependence of a galvanic cell on the current flowing through it, the graph of the dependence of the impedance of the galvanic cell on the electrical voltage is approximated (shown in Fig. 4.)

**Fig. 5:**
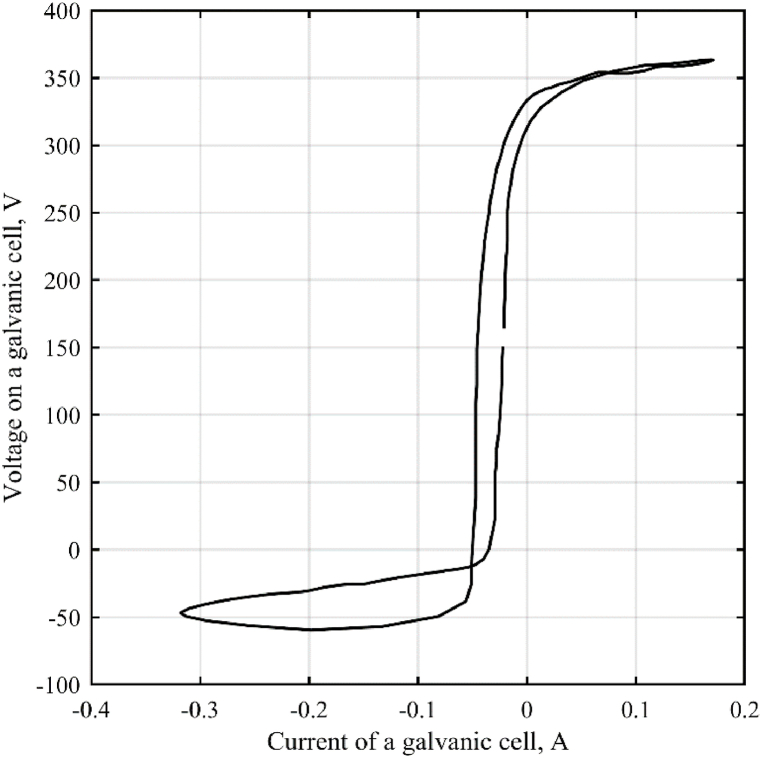
Current-voltage characteristic of a galvanic cell.

The hysteresis of the volt-ampere characteristic (Fig. 5) is the reason for the ambiguous dependence of the resistance of the galvanic cell on the current flowing through it.

The coordinates of the approximating curve for the dependence of the cell resistance on the voltage applied to it are summarized in Table 1.

**Table 1:** Values of the approximating curve.

Characteristic	Values								
Voltage, V	−50	0	50	100	150	200	250	300	350
Resistance, kOhm	0.1	5.0	8.7	12.1	13.6	12.2	0.75	0.2	0.1

The mathematical model describing the behavior of the electric circuit for the equivalent process of micro-arc oxidation will look like this:

(1) $$\left\{ \begin{array}{l} \frac{1}{C_{1}}\int I_{1}dt+R_{1}I_{1}+R_{2}\left(U_{1}\right)I_{2}=U\\ \frac{1}{C_{2}}\int I_{3}dt-R_{2}\left(U_{1}\right)I_{2}=0\\ U_{1}=U-\frac{1}{C_{1}}\int I_{1}dt\\ I_{1}=I_{2}+I_{3} \end{array}\right.\text{.}$$

## 3 Estimation of the model parameters of the equivalent electrical circuit of the MAO process

The following parameters of the equivalent circuit are subject to evaluation: the impedance of the electrolyte *R*_1_, the non-linear active resistance of the coating *R*_2_, and its equivalent capacitance *C*_2_.

To estimate the parameters of the cell equivalent circuit, we write an expression for the transfer conductivity function. From (1) follows the following expression:

(2) $$W\left(p\right)=\frac{I_{1}\left(p\right)}{U_{1}\left(p\right)}=\frac{R_{2}C_{2}p+1}{R_{1}R_{2}C_{2}p+R_{1}+R_{2}}\text{.}$$

**Fig. 6:**
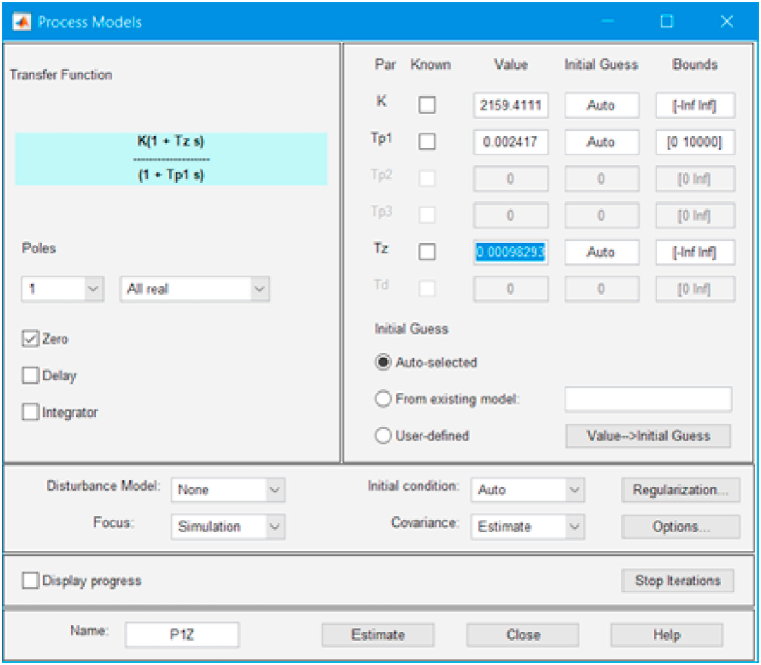
Identification results (screen dump).

We find the transfer function parameters using the MATLAB System Identification Toolbox application. As a result, the following time constants of the transfer function were obtained, shown in Fig. 6.

(3) $$T_{1}=\frac{R_{1}R_{2}C_{2}}{R_{1}+R_{2}}=0.002417\;s\text{;}$$

$$T_{2}=R_{2}C_{2}=0.0009829\;s\text{.}$$

Assuming that after breakdown the coating resistance is close to zero, the following expression is obtained to estimate *R*_1_:

(4) $${\hat{R}}_{1}=\sup\left(R_{p}\right)\text{,}$$

where **R**_*p*_ is the set of impedances of the galvanic cell formed in the breakdown areas of the coating.

If we assume that the reactance of the capacitance *C*_2_ is much greater than the maximum active resistance *R*_2_, then the estimate of *R*_2_ will be the expression

(5) $${\hat{R}}_{2}=\frac{\Delta U_{1}}{\Delta I_{1}}-{\hat{R}}_{1}\text{.}$$

Knowing the estimates ${\hat{R}}_{1}$ and ${\hat{R}}_{2}$ by expressions (3), we can find the estimate ${\hat{C}}_{2}$.

In the MATLAB environment, a program was developed for calculating the estimates of the model parameters of the equivalent electrical circuit of the MAO process. The program operation algorithm consists of the following steps.

**Fig. 7:**
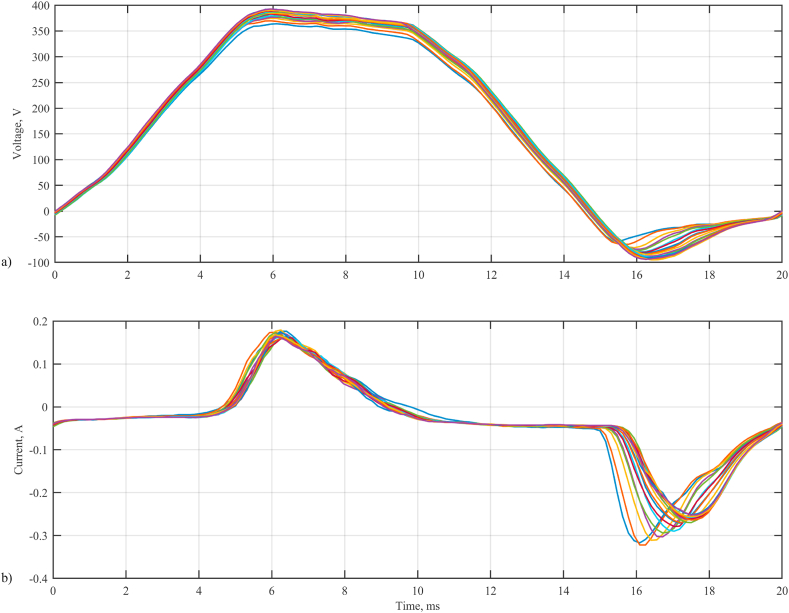
Oscillograms of voltages (a) and currents (b) of a galvanic cell.

1. During the formation of the MAO coating, 18 oscillograms of the voltage drop and current of the galvanic cell are sequentially recorded every minute (Fig. 7a and b).
2. We calculate Δ*U*_1_/Δ*I*_1_ and find the estimate ${\hat{R}}_{1}=\sup\left(R_{p}\right)$ ≈ 10 Ohm.

**Fig. 8:**
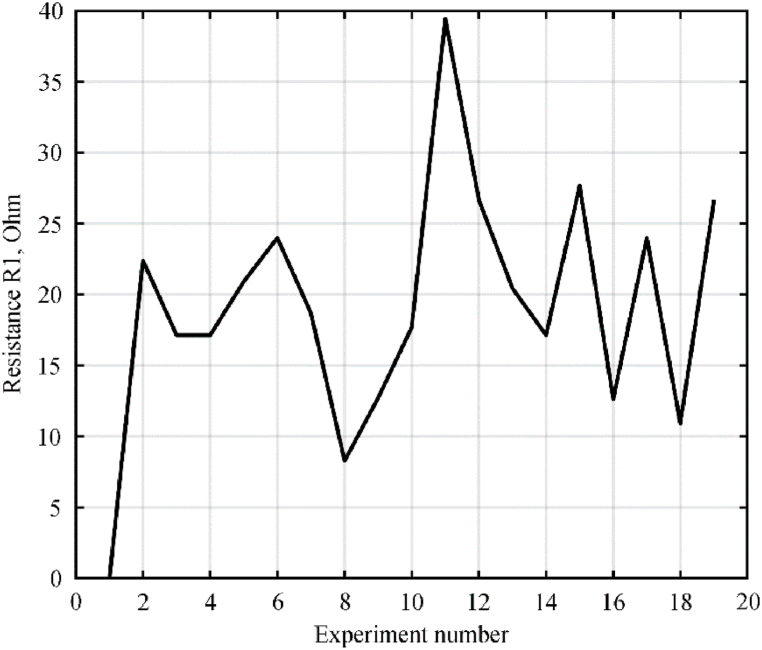
Change in resistance *R*_1_ in each experiment.

The change in the resistance *R*_1_ in different experiments is shown in Fig. 8.

**Fig. 9:**
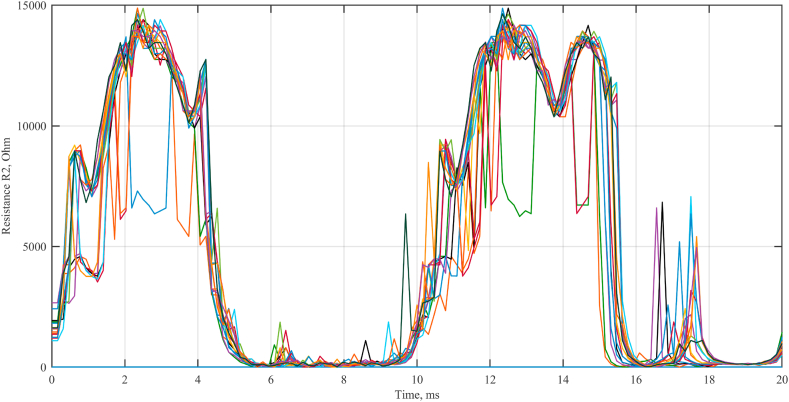
Change in resistance *R*_2_ in each experiment.

**Fig. 10:**
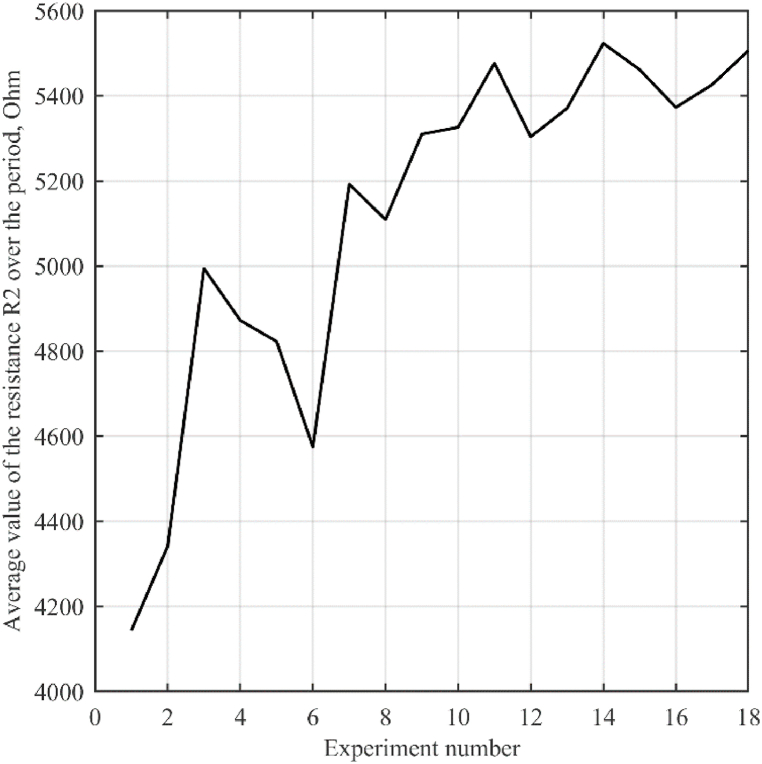
Change over the period of the average value of the resistance *R*_2_ in each experiment.

3. According to formula (5), *R*_2_ is calculated. The graph of the change in resistance *R*_2_ in various experiments is illustrated in Fig. 9.
4. We calculate the average value of the resistance *R*_2_ for the period. The change over the period of the average value of the resistance *R*_2_ is shown in Fig. 10.
5. According to formulas (3), assuming *R*_2_ ≈ 5000 Ohm, calculate *C*_2_ = 0.5 μF.

An analysis of the results of calculating the estimates of the model parameters of the equivalent electrical circuit of the MAO process showed that during the growth of the oxide coating, the value of the resistance *R*_2,_ average for the period of the electrical voltage changes noticeably. The remaining parameters of the equivalent circuit remain practically unchanged.

**Fig. 11:**
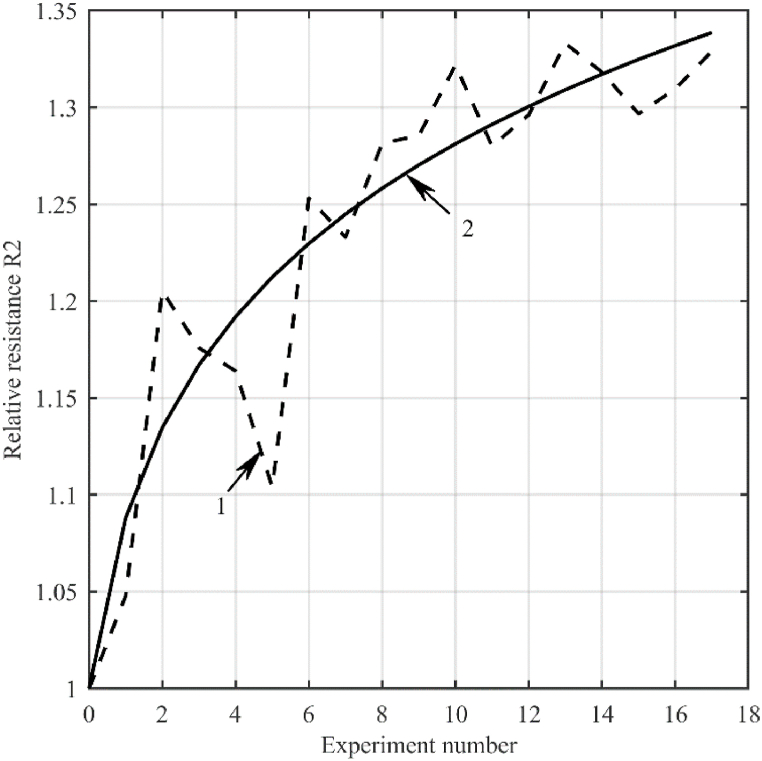
Approximation of the average value of the resistance *R*_2_ for the period of the supply voltage: 1 – experimental results; 2 – calculated data.

We will assume that the rate of growth of the MAO coating thickness is inversely proportional to its thickness, and the thickness is directly proportional to the value of the resistance *R*_2_, average for the period of the supply voltage, which we denote by *R*_*s*_. In that case, the rate of change in the relative resistance value *R*_*s*_ is approximated by the expression

(6) $$r=\frac{R_{s}}{R_{s0}}={\left(at+1\right)}^{k}\text{,}$$

where *R*_*s*0_ is the initial value of the resistance *R*_*s*_, a and k are the approximating coefficients that can be found by minimizing the discrepancy between the experimental and calculated data. Fig. 11 shows the approximation results obtained with *a* = 1.6542 and *k* = 0.0865.

The main characteristics of the linearized model of the MAO process were calculated. To do this, the model was presented in the state space for the idle (no coating breakdown) and short circuit (coating breakdown) modes.

(7) $$\left\{ \begin{array}{l} \frac{dx}{dt}=Ax+BU\\ y=Cx+DU \end{array}\right.\text{,}$$

where $x={\left(\begin{array}{cc} I_{1} & I_{2} \end{array}\right)}^{T}$; *y* = *I*_1_;

$$A=\left(\begin{array}{cc} -\frac{C_{1}+C_{2}}{C_{1}C_{2}R_{1}} & \frac{1}{C_{2}R_{1}}\\ \frac{1}{C_{2}R_{2}} & -\frac{1}{C_{2}R_{2}} \end{array}\right);\;B=\left(\begin{array}{l} \frac{1}{R_{1}}\\ 0 \end{array}\right)\text{;}$$

$$C=\left(\begin{array}{cc} 1 & 0 \end{array}\right);\;D=0;\;R_{2}=f\left(U_{1}\right)\text{.}$$

**Fig. 12:**
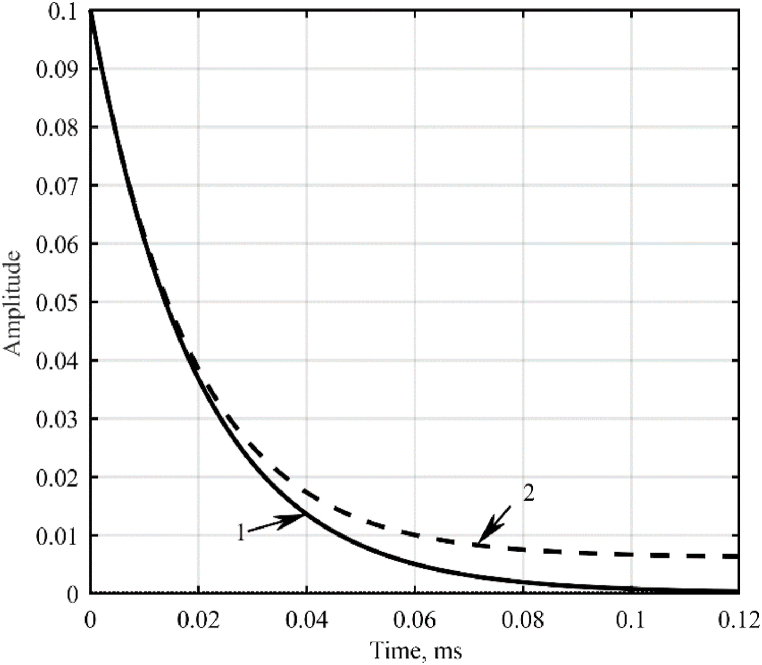
Impulse transient responses of a galvanic cell model: 1 – idle mode; 2 – short-circuit mode.

**Fig. 13:**
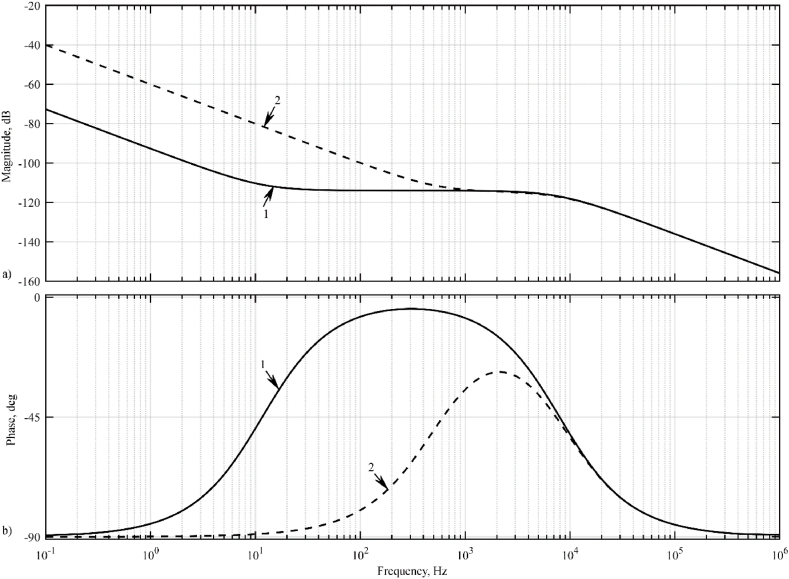
Logarithmic amplitude-frequency response (a) and phase-frequency response (b) of the galvanic cell model: 1 – idle mode; 2 – short-circuit mode.

Fig. 12 shows the impulse response of the model, Fig. 13 shows its logarithmic amplitude-frequency response and phase-frequency response characteristics.

The model is stable, since the roots of the characteristic equation are positive, the Model is controllable and observable, since the ranks of the controllability and observability matrices are equal to the order of the system, however, the model is ill-conditioned, which can lead to significant errors in its parametric identification.

## 4 Statement of the problem of identifying the model parameters of the electric equivalent circuit of the MAO process

Estimation of the model parameters of the equivalent electrical circuit of the MAO process showed that the main variable parameter of the model of the electrical circuit is the impedance of the cell coating. At the same time, when calculating the parameters of the equivalent circuit from experimental oscillograms, significant identification errors are observed due to the poor model conditionality.

To reduce identification errors, it is proposed to use neural networks in the framework of reservoir computing to solve the considered identification problem [[27], [28], [29]].

The main idea of reservoir computing is to use a dynamic neural network as a reservoir that is filled with displays of various states of the original system obtained using this network, while filling the reservoir can occur both purposefully and randomly. The data filling the reservoir is read by a static neural network, which, according to the state of the reservoir, solves the problem of calculating the required parameters [30].

We formulate the initial formulation of the reservoir calculations problem of the MAO model parameters in the following formulation.

The arrays of experimental oscillograms (Fig. 4) of the voltage

(8) $$U=\left[\begin{array}{cccc} U_{1}\left(t_{1}\right) & U_{1}\left(t_{2}\right) & \ldots & U_{1}\left(t_{n}\right)\\ U_{2}\left(t_{1}\right) & U_{2}\left(t_{2}\right) & \ldots & U_{2}\left(t_{n}\right)\\ & \vdots & & \\ U_{m}\left(t_{1}\right) & U_{m}\left(t_{2}\right) & \ldots & U_{m}\left(t_{n}\right) \end{array}\right]$$

and current

(9) $$I=\left[\begin{array}{cccc} I_{1}\left(t_{1}\right) & I_{1}\left(t_{2}\right) & \ldots & I_{1}\left(t_{n}\right)\\ I_{2}\left(t_{1}\right) & I_{2}\left(t_{2}\right) & \ldots & I_{2}\left(t_{n}\right)\\ & \vdots & & \\ I_{m}\left(t_{1}\right) & I_{m}\left(t_{2}\right) & \ldots & I_{m}\left(t_{n}\right) \end{array}\right]$$

obtained at fixed equidistant time intervals *t*_1_, *t*_2_, …*t*_*n*_ in each of m experiments carried out at regular time intervals of 1 min, as well as the vector for estimating the parameters of the MAO model **Θ** = [*R*_1_, *R*_2_, *C*_2_]^*T*^, obtained from the results of processing arrays (8) and (9), are given.

Arrays (8) and (9) form a training sample

(10) $$P_{d}=\left[U,I\right];\;T_{d}=I\text{,}$$

used to train a dynamic neural network. The learning result maps the arrays **P**_**d**_, **T**_**d**_ to the array of synaptic and weight coefficients of the dynamic neural network **W**_**d**_.

(11) $$\left[P_{d},T_{d}\right]\overset{\text{learning}}{\to }W_{d}\text{.}$$

The second static neural network based on the training sample **W**_**d**_, composed of the array of synaptic coefficients of the trained dynamic neural network and the model parameter estimation vector **Θ**

(12) $$P_{c}=W_{d};\;T_{c}=\Theta$$

calculates a new estimate of the model parameters $\hat{\Theta }$.

(13) $$\left[W_{k},\Theta \right]\overset{\text{learning}}{\to }\hat{\Theta }\text{.}$$

Taking into account the powerful approximating capabilities of neural networks, one should expect high accuracy of parametric identification.

## 5 Parametric identification algorithm

The following algorithm is proposed for calculating the average value of the resistance *R*_2_ over the period of the supply voltage.

1. From the experimental oscillograms shown in Fig. 1, training and control samples are formed.
2. On the sample **P**_**d**_, **T**_**d**_ (10), a dynamic neural network is trained and, as a result of training, a matrix of its synaptic coefficients **W**_**d**_ (11) is formed.
3. A new training sample is formed to train the second static neural network (12).
4. The second static network is trained on the sample (12) and the estimate of the parameter vector $\hat{\theta }$ of the model (1) is calculated in each of *m* experiments.
5. Experimental oscillograms (2) that did not participate in training experiments are fed to the input of the dynamic neural network, and the vector of synaptic coefficients **w**_**r**_ for the current state of the object is evaluated.
6. The calculated coefficients are fed to the input of the next trained static neural network, then the vector of model parameters is estimated to describe the current state of the real object $\hat{\theta }$.

## 6 Parametric identification of a non-linear object model

Parametric identification was carried out in accordance with the proposed algorithm:

1. 15 experimental oscillograms were used to obtain the training sample (10). Oscillograms 3, 9 and 15 made up the control sample.
2. Sample (10) was used to train a three-layer feedforward neural network with 2 neurons and a tangential activation function in the first layer and with one neuron and linear activation functions in the second and third layers. The number of synaptic coefficients and neural network bias functions are summarized in Table 2.

**Table 2:** Number of synaptic coefficients and bias functions.

Layers/coefficients	Synaptic coefficients	Displacement functions	Activation functions
First layer	8	2	Tangential
Second layer	2	1	Linear
Third layer	1	1	Linear

The training sample is a 15 × 15 matrix consisting of 15 rows for the number of experiments and 15 columns for the number of synaptic coefficients and bias functions. The network was trained according to the Levenberg-Marquardt method with Bayesian regularization. The maximum value of the current calculation error did not exceed 0.1 mA.

3. From the obtained matrix of synaptic coefficients of the dynamic neural network, a new training sample (12) is formed to train the second static neural network.
4. The training set (12) was used to train the second neural network, which was chosen as the radial basic network, which provides a zero-learning error.
5. The experimental values of the current and voltage of the galvanic cell from the control sample were fed to the input of the neural network, then the vector of its synaptic coefficients and bias functions were calculated.
6. The calculated parameter vectors of the first neural network were fed to the input of the trained second static neural network, and the estimate of the parameter vector of the real object model $\hat{\theta }$ was calculated (Table 3).

**Table 3:** Evaluation of identification results.

Parameters	*r* (3)	*r* (9)	*r* (15)
Estimated	1.1347	1.2583	1.3173
Identification	1.1347	1.2583	1.3173

It can be noted that the calculated data of the relative resistance value *r*, obtained by expression (6), coincide exactly with the data obtained as a result of identification.

## 7 Conclusions

The paper solves the problems of parametric identification of a nonlinear model of an object using the example of a mathematical model of the micro-arc oxidation process, which is advisable to use in the development of an intelligent hardware-software complex for obtaining protective coatings with desired properties by the micro-arc oxidation method. The following main results have been obtained:

1. The problem of parametric identification of models of nonlinear objects is formulated, which boils down to mapping experimentally obtained input and output variables of an object into an estimate of its parameters using two neural networks.
2. An algorithm for parametric identification, the essence of which is to conduct an experiment on a real object, forming training and control samples based on the results of the experiment, sequentially training neural networks and calculating, using trained networks, estimates of the parameters of a nonlinear model from experimental data, was developed.
3. A combination of two neural networks is proposed, the distinctive feature of which is the supply of synaptic coefficients of the first neural network to the input of the second neural network during training and subsequent work.
4. In such a network, the mapping of experimental data to model parameters is carried out in the following sequence: experimental data are compared with the synaptic coefficients of the first neural network, and then they are fed to the inputs of the second neural network, the output of which contains the desired parameters of the nonlinear model.
5. Experimental verification of the proposed method of neural network parametric identification using the micro-arc oxidation process as an example, considered in detail in Refs. [22,23], showed that the standard deviation of current and voltage from the nominal values does not exceed ±4%.
6. Taken into account the good approximating ability of neural networks, the proposed algorithm and neural networks can be considered as an effective identification method.

Thus, the results of the study make it possible to carry out current monitoring and control of the microarc oxidation process with subsequent automation, which ensures a significant increase in its technical and economic indicators.

## Funding

The work was supported by the grant of the Ministry of Science and Higher Education of the Russian Federation, Russia № 1022041100284-5-2.3.1. «Fundamentals of the digital twin of the technological process of forming oxide coatings with specified properties by microarc oxidation» (FSGE-2023-0005).

## Author contribution statement

Anatoliy Semenov: conceived and designed the experiments; analyzed and interpreted the data; wrote the paper.

Ekaterina Pecherskaya: conceived and designed the experiments; analyzed and interpreted the data; wrote the paper.

Pavel Golubkov & Sergey Gurin: performed the experiments; analyzed and interpreted the data.

Dmitriy Artamonov: analyzed and interpreted the data.

Yuliya Shepeleva: performed the experiments.

## Data availability statement

Data will be made available on request.

## Declaration of competing interest

The authors declare that they have no known competing financial interests or personal relationships that could have appeared to influence the work reported in this paper.
